# Reversible Subacute Parkinsonism and Cognitive Decline in a Patient With Acquired Hepatocerebral Degeneration: A Case Report

**DOI:** 10.7759/cureus.93431

**Published:** 2025-09-28

**Authors:** Shoji Kikui, Daisuke Danno, Takao Takeshima

**Affiliations:** 1 Neurology, Tominaga Hospital, Osaka, JPN

**Keywords:** acquired hepatocerebral degeneration, cognitive decline, hyperammonemia, parkinsonism, reversibility

## Abstract

Acquired hepatocerebral degeneration (AHD) is a rare neurological disorder associated with chronic liver disease and portosystemic shunting. It is characterized by parkinsonism, cognitive impairment, and bilateral hyperintensities in the globus pallidus on T1-weighted imaging. Although traditionally considered irreversible, recent reports suggest that AHD may be partially reversible with early intervention. We describe the case of a 71-year-old Japanese man with a history of chronic alcohol use and liver dysfunction who developed subacute parkinsonism and cognitive decline, initially preceded by depressive symptoms. He was treated for major depressive disorder before neurological signs emerged. Laboratory testing revealed macrocytic anemia, mild hepatic dysfunction, and hyperammonemia. T1-weighted imaging showed bilateral hyperintensities in the globus pallidus, despite a normal serum manganese level. A diagnosis of AHD was made based on these findings. Conservative treatment with IV branched-chain amino acids and oral lactulose led to rapid and marked clinical improvement. This case highlights the potential reversibility of early-stage AHD and emphasizes the importance of recognizing psychiatric symptoms, such as depression, as possible early manifestations. Recent literature has documented similar cases of AHD initially misdiagnosed as primary psychiatric disorders, underscoring the need for careful diagnostic evaluation when basal ganglia abnormalities are detected on MRI. Early recognition and metabolic correction may help prevent irreversible neurological damage and improve patient outcomes.

## Introduction

Acquired hepatocerebral degeneration (AHD), first described by Victor and Adams in 1965, is a progressive neurological disorder associated with chronic liver disease and portosystemic shunting [1]. It is characterized by movement disorders, most notably parkinsonism, along with cognitive and psychiatric symptoms, occurring in the absence of overt hepatic encephalopathy (HE) [1,2]. A hallmark radiological feature is bilateral hyperintensity of the globus pallidus on T1-weighted MRI, typically attributed to manganese (Mn) accumulation in the basal ganglia [1,3].

Although AHD is considered rare, the reported prevalence of parkinsonism in patients with cirrhosis varies widely. Earlier reviews estimated a prevalence of approximately 2% [1], whereas more recent studies have suggested considerably higher rates [4], with one prospective study reporting parkinsonism in 52% of cirrhotic patients [5].

At present, no universally accepted diagnostic criteria for AHD exist. However, a practical framework has been proposed that includes three core features: (1) the presence of chronic liver disease or portosystemic shunting, which may lead to Mn accumulation; (2) neurological symptoms; and (3) bilateral globus pallidus hyperintensity on T1-weighted imaging [3]. Traditionally regarded as a chronic and largely irreversible condition, AHD has more recently been reported to show partial reversibility, particularly when diagnosed early and managed with metabolic interventions such as ammonia-lowering therapy [4,5].

Here, we describe a case of AHD in a patient with chronic alcoholic liver disease who presented with subacute parkinsonism and cognitive decline. His neurological symptoms improved markedly after conservative treatment. This case underscores the importance of early recognition of AHD, including potential psychiatric manifestations such as depression [6], and highlights the possibility of reversibility in the early stages of the disease.

## Case presentation

A 71-year-old Japanese man presented with gait disturbance, characterized by difficulty initiating steps and a tendency to fall, which had led to an L1 compression fracture. His medical history included a cholecystectomy at age 60 years and atrial fibrillation diagnosed at age 70 years, for which dabigatran (220 mg/day) had been initiated. The patient had no history of smoking. He had consumed approximately 60-70 g of ethanol daily for 30 years but stopped drinking after the fall without developing withdrawal symptoms.

At the time of the fall and L1 compression fracture, he was alert and showed no evidence of cognitive impairment. The fall may have reflected early parkinsonian gait disturbances. Several months later, he developed apathy and a depressed mood and was diagnosed with major depressive disorder at a psychiatric clinic. Sertraline was prescribed at 25 mg/day and titrated to 100 mg/day. After four months of sertraline treatment, his depressive symptoms had slightly improved; however, he developed bradykinesia, shuffling gait, and masked facies and was referred for evaluation of suspected Parkinson’s disease. He was subsequently seen at the Department of Neurology, Tominaga Hospital.

On neurological examination, he was alert with a masked face, mild upward gaze limitation, and rigidity in all four limbs. His gait was short-stepped with forward flexion. Muscle strength was preserved according to the Medical Research Council (MRC) scale (MRC grade 5/5 bilaterally), tremors were absent, and deep tendon reflexes were normal. Constipation was noted as an autonomic symptom. He was oriented, able to converse, and showed no evidence of aphasia, apraxia, or agnosia, indicating preserved cortical function at that time.

Laboratory testing revealed macrocytic anemia, reduced red blood cell count and hematocrit, and increased mean corpuscular volume and mean corpuscular hemoglobin, accompanied by thrombocytopenia. Mild hepatic dysfunction was also noted, with elevated aspartate aminotransferase, alkaline phosphatase, and γ-glutamyl transpeptidase levels. The activated partial thromboplastin time was prolonged, which was attributed to ongoing dabigatran therapy. Detailed laboratory findings at the initial outpatient visit and at admission are summarized in Table 1.

**Table 1:** Laboratory findings at the initial outpatient visit and at hospital admission ALP, alkaline phosphatase; ALT, alanine aminotransferase; APTT, activated partial thromboplastin time; AST, aspartate aminotransferase; γ-GT, γ-glutamyltransferase; Ht, hematocrit; MCH, mean corpuscular hemoglobin; MCV, mean corpuscular volume; PLT, platelet

Parameter	Initial outpatient visit	At admission	Reference range
RBC (× 10^4^/μL)	348	375	435-555
Ht (%)	37.7	38.6	40.7-50.1
MCV (fL)	108.3	102.9	83.6-98.2
MCH (pg)	36.5	36	27.5-33.2
PLT (× 10^4^/μL)	11.1	12.2	13.7-16.8
AST (IU/L)	43	37	13-33
ALT (IU/L)	27	13	8-42
ALP (IU/L)	428	275	115-359
γ-GT (IU/L)	82	74	10-47
Ammonia (μg/dL)	-	323	12-66
APTT (seconds)	47	51.4	24-40

**Figure 1:**
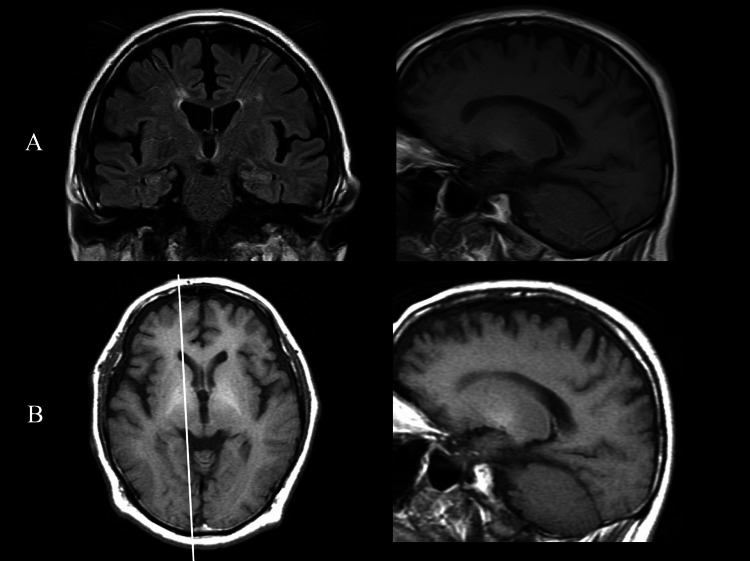
Brain MRI (A) At the initial outpatient visit, coronal FLAIR imaging revealed mild diffuse cerebral atrophy and subtle periventricular ischemic changes. Sagittal T1-weighted imaging showed hyperintensity in the globus pallidus. (B) At hospital admission, axial T1-weighted imaging demonstrated increased signal intensity in the globus pallidus (white arrow), with the corresponding sagittal T1-weighted image confirming the hyperintensity more clearly compared with the previous scan. FLAIR, fluid-attenuated inversion recovery

Thyroid function, vitamin B12, and folate levels were within the normal range. Serum copper and Mn levels were also normal. Brain MRI revealed mild diffuse cerebral atrophy and subtle periventricular ischemic changes on fluid-attenuated inversion recovery (FLAIR) imaging. Although axial T1-weighted images were not obtained, sagittal T1-weighted imaging showed faint bilateral hyperintensities in the globus pallidus (Figure 1A), raising the possibility of AHD in the setting of chronic liver dysfunction. Iodine-123 metaiodobenzylguanidine scintigraphy demonstrated preserved cardiac sympathetic innervation.

Sertraline-induced drug-related parkinsonism was also considered. After the initial outpatient visit, the sertraline dose was reduced to 50 mg/day. At the follow-up one week later, the parkinsonism had slightly worsened, and the dose was further reduced to 25 mg/day. One week later, his parkinsonism progressed further, and sertraline was discontinued while a levodopa/decarboxylase inhibitor (DCI) was initiated at 100 mg/10 mg/day. Over the following two weeks, despite these adjustments, his condition deteriorated, leading to disorientation, inability to walk, and emergency hospitalization. Neither gradual dose reduction nor discontinuation of sertraline halted progression, and treatment with levodopa/DCI did not produce any clinical benefit.

On admission, the patient was febrile but hemodynamically stable. His level of consciousness was impaired (Glasgow Coma Scale (GCS) score, 12; E3, V3, M6), and he scored 22 out of 30 on the Mini-Mental State Examination (MMSE), with points deducted for time orientation (-3), place orientation (-1), attention and calculation (-2), and delayed recall (-2). Neurological examination revealed bilateral rigidity, more pronounced on the left, along with depressed tendon reflexes and mild proximal weakness of both lower limbs (MRC grade 4/5), without tremor or ataxia.

Laboratory testing showed persistent macrocytic anemia, mild thrombocytopenia, and mild hepatic dysfunction, consistent with previous findings. Serological tests were negative for hepatitis B surface antigen, hepatitis C virus antibody, Treponema pallidum hemagglutination assay, rapid plasma reagin, and HIV antibody. Lactate, pyruvate, and vitamin B1 levels were within normal limits. However, the blood ammonia level was markedly elevated. EEG at admission revealed diffuse slow waves without triphasic or epileptiform discharges. Because the patient had no history of epilepsy or use of antiepileptic drugs, and his clinical course improved with ammonia-lowering therapy, a follow-up EEG was not performed.

**Figure 2:**
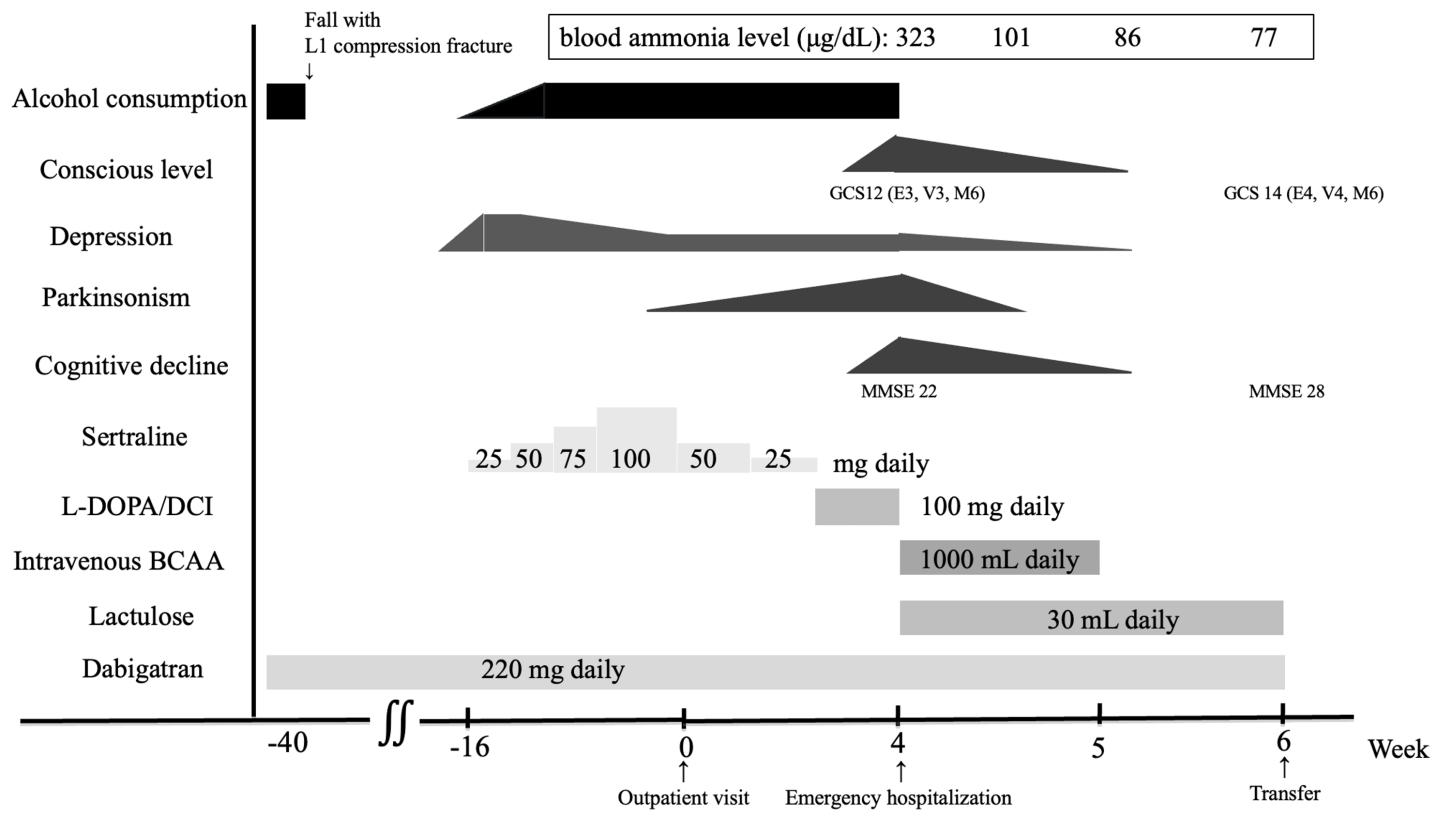
Clinical course Time-course diagram illustrating therapeutic interventions, level of consciousness, MMSE score, and serum ammonia levels over time. BCAA, branched-chain amino acid; DCI, decarboxylase inhibitor; GCS, Glasgow Coma Scale; MMSE, Mini-Mental State Examination

The repeat MRI demonstrated increased T1 signal intensities in the globus pallidus compared with the previous scan (Figure 1B). Given the presence of progressive parkinsonism, cognitive dysfunction, chronic hepatic dysfunction, hyperammonemia, and characteristic MRI findings, AHD was diagnosed. Treatment was initiated with IV branched-chain amino acid (BCAA) solution (1000 mL/day), commonly used for HE, and oral lactulose (30 mL/day). His clinical course and treatment are summarized in Figure 2.

His serum ammonia levels progressively decreased after treatment was initiated, accompanied by marked improvements in consciousness and motor symptoms. Abdominal ultrasonography revealed an irregular liver margin and heterogeneous parenchyma. Abdominal computed tomography showed hepatic atrophy, surface irregularity, and suspected gastrorenal shunting, suggesting underlying cirrhosis with portosystemic shunting despite the absence of portal hypertension.

He received IV BCAA therapy for one week and was subsequently transferred to a specialized center for further management of chronic liver disease. Improvement in parkinsonism began four days after treatment initiation, and by one week, his level of consciousness, depressive symptoms, and cognitive function had also improved. At the time of transfer, two weeks after admission, his GCS score was 14 (E4, V4, M6), and his MMSE score was 28 (-1 in time orientation and -1 in delayed recall). After his consciousness improved, he disclosed that he had secretly resumed alcohol consumption at the time of his diagnosis of major depressive disorder.

## Discussion

AHD is a rare neurological disorder associated with chronic liver disease and portosystemic shunting, typically presenting with parkinsonism, cognitive dysfunction, and characteristic bilateral hyperintensities of the globus pallidus on T1-weighted imaging [1,3,6]. Although no universally accepted diagnostic criteria currently exist, Nascimento et al. proposed a practical framework consisting of three core features: (1) chronic liver disease or portosystemic shunting; (2) neurological manifestations such as extrapyramidal signs or cognitive impairment; and (3) bilateral globus pallidus hyperintensities on T1-weighted imaging [3]. Our patient fulfilled all three criteria, supporting a clinical diagnosis of AHD.

Differentiating AHD from HE is critical, as their clinical features, course, and prognosis differ substantially. HE typically presents acutely or with fluctuating symptoms, and it responds well to ammonia-lowering therapies such as lactulose or rifaximin; triphasic waves are frequently observed on EEG [7,8]. In contrast, AHD progresses subacutely over weeks to months, is characterized primarily by parkinsonism, lacks triphasic waves on EEG, and demonstrates specific MRI changes in the basal ganglia [4,7]. In our case, the EEG showed diffuse slowing without epileptiform activity, and there was no history of epilepsy or prior antiepileptic drug use. Therefore, the improvement in consciousness was attributed to metabolic correction rather than to the resolution of seizure activity.

Table 2 summarizes the diagnostic differences. Although hyperammonemia was present in our patient, the predominant features of subacute parkinsonism, cognitive decline, and bilateral globus pallidus hyperintensities favored a diagnosis of AHD over HE.

**Table 2:** Clinical and diagnostic differences between AHD and HE AHD, acquired hepatocerebral degeneration; BCAA, branched-chain amino acid; DWI, diffusion-weighted imaging; FLAIR, fluid-attenuated inversion recovery; HE, hepatic encephalopathy; Mn, manganese Data derived from previous studies [1-8]. Table credit: Shoji Kikui

Feature	AHD	HE
Pathophysiology [1,2,3,7]	Chronic exposure of the brain to neurotoxins (especially Mn) because of liver dysfunction or portosystemic shunting	Accumulation of ammonia and other neurotoxins due to acute or chronic liver failure
Onset and course [4,7]	Subacute to chronic, insidious progression over weeks to months	Acute or fluctuating course, often within hours to days
Main clinical features [1,4,7]	Parkinsonism, gait disturbance, and cognitive decline	Altered mental status, confusion, lethargy, stupor, or coma
Consciousness impairment [4,7]	Gradual and mild-to-moderate impairment	Rapid onset; may progress to deep coma
EEG findings [7]	Diffuse slowing; triphasic waves are usually absent	Diffuse slowing with typical triphasic waves
MRI findings [1,3,4,7]	Bilateral hyperintensities in the globus pallidus on T1-weighted imaging (nonspecific but characteristic)	May show diffuse white matter changes or cortical abnormalities on FLAIR/DWI; occasional T1 hyperintensity in chronic cases
Serum ammonia [7,8]	May be elevated, but not the primary pathogenic factor	Typically elevated and correlates with clinical severity
Serum Mn [2,3]	Normal or mildly elevated; does not reflect brain Mn load	Usually normal
Response to treatment [4,7]	Partial improvement possible with conservative therapy (e.g., BCAA and lactulose); may require a liver transplant in advanced stages	Reversible with ammonia-lowering therapy (e.g., lactulose and rifaximin)
Prognosis [4,5,7]	May improve if diagnosed early; often considered partially irreversible	Good with appropriate treatment; depends on liver function

AHD has long been considered a progressive and largely irreversible condition; however, recent reports have highlighted the potential for partial or even marked reversibility, particularly when metabolic derangements are corrected early [4,5]. In this context, “early stage” generally refers to the period within several months after the onset of neurological symptoms, before irreversible neuronal damage occurs. Consistent with this definition, in our case, treatment was initiated approximately four months after the onset of parkinsonism, and clinical improvement was observed within one week. Conservative therapy with IV BCAA and oral lactulose resulted in rapid normalization of serum ammonia levels (323 → 77 µg/dL), complete resolution of parkinsonism, and improvement in the MMSE score from 22 to 28 points. This outcome supports the emerging view that early-stage AHD may be reversible if it is diagnosed promptly and treated appropriately.

Furthermore, growing evidence suggests that AHD and HE may not be entirely separate entities but instead exist along a neurotoxic spectrum related to hepatic dysfunction [4]. Both disorders share overlapping features, such as cognitive impairment, elevated ammonia levels, and, in some cases, MRI abnormalities. In this case, the coexistence of hyperammonemia and parkinsonism, along with the favorable response to ammonia-lowering therapy, supports the concept that AHD and HE may represent phenotypic variants within a shared pathophysiological continuum. Sertraline-induced parkinsonism was initially suspected; however, symptoms worsened despite discontinuation of sertraline and showed no response to levodopa. In contrast, marked improvement occurred after BCAA infusion and lactulose treatment, indicating that the parkinsonism was due to AHD rather than a drug-induced adverse effect.

It is also important to note that serum Mn levels were within normal limits in our patient. However, this did not preclude the diagnosis of AHD. Prior studies have demonstrated that serum Mn concentration does not reliably reflect the extent of Mn accumulation in the brain and that characteristic MRI findings and clinical symptoms should remain the cornerstone of diagnosis [2,6].

This case further demonstrates that psychiatric symptoms, such as depression, may precede overt neurological signs in AHD, consistent with recent reports of misdiagnosis as a primary psychiatric disorder [6]. Given that subtle MRI findings, such as globus pallidus T1 hyperintensities, may initially be overlooked, clinicians should maintain a high index of suspicion for AHD in patients with chronic liver disease and treatment-resistant psychiatric symptoms.

## Conclusions

This case demonstrates that AHD, traditionally considered an irreversible condition, can show marked clinical improvement when recognized early and promptly treated with metabolic interventions. The patient’s rapid recovery of both cognitive function and parkinsonian symptoms following BCAA infusion and lactulose therapy underscores the importance of maintaining a high index of suspicion for this condition in individuals with chronic liver disease who present with subacute neurological or psychiatric symptoms. Careful evaluation of brain MRI findings, even when subtle, and early differentiation from HE are essential for initiating appropriate treatment and preventing permanent neurological sequelae. This case also highlights that depressive symptoms may precede overt neurological signs, emphasizing the need for multidisciplinary collaboration among neurology, psychiatry, and hepatology in the diagnostic process.

## References

[1] Shin HW, Park HK (2017). Recent updates on acquired hepatocerebral degeneration. Tremor Other Hyperkinet Mov (N Y).

[2] Mehkari Z, Mohammed L, Javed M (2020). Manganese, a likely cause of 'Parkinson's in cirrhosis', a unique clinical entity of acquired hepatocerebral degeneration. Cureus.

[3] Nascimento H, Malaquias MJ, Pinto CM (2023). Trace element imbalances in acquired hepatocerebral degeneration and changes after liver transplant. Biology (Basel).

[4] Malaquias MJ, Pinto CM, Ramos C (2020). Acquired hepatocerebral degeneration and hepatic encephalopathy: one or two entities?. Eur J Neurol.

[5] Apetauerova D, Hildebrand P, Scala S (2021). A prospective study of the prevalence of Parkinsonism in patients with liver cirrhosis. Hepatol Commun.

[6] Klos KJ, Ahlskog JE, Josephs KA, Fealey RD, Cowl CT, Kumar N (2005). Neurologic spectrum of chronic liver failure and basal ganglia T1 hyperintensity on magnetic resonance imaging: probable manganese neurotoxicity. Arch Neurol.

[7] Romeiro FG, Américo MF, Yamashiro FS, Caramori CA, Schelp AO, Santos AC, Silva GF (2011). Acquired hepatocerebral degeneration and hepatic encephalopathy: correlations and variety of clinical presentations in overt and subclinical liver disease. Arq Neuropsiquiatr.

[8] Butterworth RF (2011). Hepatic encephalopathy: a central neuroinflammatory disorder?. Hepatology.

